# Incidental Carcinoma after Cholecystectomy for Benign Disease of the Gallbladder: A Meta-Analysis

**DOI:** 10.3390/jcm9051484

**Published:** 2020-05-14

**Authors:** Jung-Soo Pyo, Byoung Kwan Son, Hyo Young Lee, Il Whan Oh, Kwang Hyun Chung

**Affiliations:** 1 Department of Pathology, Daejeon Eulji University Hospital, Eulji University School of Medicine, Daejeon 35233, Korea; jspyo@eulji.ac.kr; 2 Department of Internal Medicine, Nowon Eulji University Hospital, Eulji University School of Medicine, Seoul 01830, Korea; 2hyo0@eulji.ac.kr (H.Y.L.); 20180121@eulji.ac.kr (I.W.O.); kh.chung@eulji.ac.kr (K.H.C.)

**Keywords:** gallbladder, incidental carcinoma, incidence, prognosis, meta-analysis

## Abstract

This study aimed to determine the incidence and the prognosis of incidental carcinoma of the gallbladder (IGBC) after cholecystectomy through a meta-analysis. This meta-analysis included 51 studies and 436,636 patients with cholecystectomy. The incidence rate of IGBC after cholecystectomy was 0.6% (95% confidence interval (CI) 0.5–0.8%). The incidence rate of recent studies was not significantly different from those of past studies. The mean age and female ratio of the IGBC subgroup were not significantly different from those of the overall patient group. The estimated rates of IGBC were 13.0%, 34.1%, 39.7%, 22.7%, and 12.5% in the pTis, pT1, pT2, pT3, and pT4 stages, respectively. Patients with IGBC had a favorable overall survival rate compared to patients with non-IGBC (hazard ratio (HR) 0.574, 95% CI 0.445–0.739). However, there was no significant difference of disease-free survival between the IGBC and non-IGBC subgroups (HR 0.931, 95% CI 0.618–1.402). IGBC was found in 0.6% of patients with cholecystectomy. The prognosis of patients with IGBC was favorable compared to those with non-IGBC. In the pathologic examination after cholecystectomy for benign diseases, a sufficient examination for histology should be guaranteed to detect IGBC.

## 1. Introduction

Gallbladder cancer (GBC), which is the most common biliary tract carcinoma, has nonspecific signs and symptoms and is sometimes indistinguishable from benign disease of the gallbladder (GB) [1]. Representative radiologic findings of GBC include wall thickening or mass-forming. The most common finding of radiology is wall thickening [2]. However, because the GB wall may be thickened by coexisting benign disease, such as cholelithiasis, the masking of GBC can occur. In addition, these radiologic findings of GBC are not specific and may be overlapped with benign diseases of the GB, such as focal adenomyomatosis or GB polyps [2]. Therefore, GBCs cannot be detected in preoperative radiologic examinations. GBCs, which are identified in the pathologic examination after cholecystectomy, are defined as incidental GBC (IGBC). 

The incidence rate of IGBC after cholecystectomy was found to range up to 2.9% and this varied according to reports [3,4,5,6,7,8,9,10,11,12,13,14,15,16,17,18,19,20,21,22,23,24,25,26,27,28,29,30,31,32,33,34,35,36,37,38,39,40,41,42,43,44,45,46,47,48,49,50,51,52,53]. However, IGBC may be up to 50% of all GBCs [54]. GBC is the fifth most common malignant tumor of the gastrointestinal tract, with an incidence of 0.8–1.2% [55]. In healthy populations, the prevalence of cholecystectomy, which is one of the most common surgeries performed, were 1.3%, 2.9%, and 11.1% in Taiwan, Brazil, and German, respectively [10,56]. The different prevalences of cholecystectomy can affect the detection rate of IGBC. Cholelithiasis, which is an important cause for cholecystectomy, are affecting 10% to 15% of the adult population of developed countries [57]. In the diagnosis of GBC, pathologic examinations after cholecystectomy are essential for the detection of IGBC. Cholelithiasis is the most common risk factor of GBC [5]. The risk factors of IGBC include old age, female, cholelithiasis, and obesity [1]. Preoperatively, the suspicious clinical and radiologic findings of IGBC are not specific, and the risk factors of IGBC and non-IGBC are overlapped [2]. The performance rates of pathologic examinations after cholecystectomy may be varied according to the country and insurance policy [25]. If the pathologic examinations after cholecystectomy were not performed in every case, the incidence rate of IGBC might be overestimated or underestimated. When this information for IGBC is accurate, further studies for the treatment of IGBC and predictions of the prognosis will be possible. In the present study, we aimed to elucidate the incidence rate of IGBC and the prognosis of IGBC through a meta-analysis. In addition, to obtain the clinicopathologic characteristics of IGBC, the mean age and compositions of the female ratio and pT stage were analyzed and compared to those of GBC overall.

## 2. Materials and Methods

### 2.1. Published Study Search and Selection Criteria

The literature search was performed using the PubMed databases through 31 July 2019. The search was performed using the following keywords: “cholecystectomy”, “unsuspected or incidental”, and “cancer or carcinoma or malignant or malignancy.” The titles and abstracts of the searched articles were primarily screened for exclusion. Literature or systematic review articles were also screened to find additional eligible studies. The inclusion and exclusion criteria were as follows: (1) studies for the cholecystectomy by IGBCs in human samples were included and (2) non-original articles, such as case reports or review articles, were excluded.

### 2.2. Data Extraction

For the meta-analysis, data were extracted from the eligible studies [3,4,5,6,7,8,9,10,11,12,13,14,15,16,17,18,19,20,21,22,23,24,25,26,27,28,29,30,31,32,33,34,35,36,37,38,39,40,41,42,43,44,45,46,47,48,49,50,51,52,53] as follows: the first author’s name, study location, study year, type of study and initial surgery, number of patients analyzed, patients’ age and sex, tumor stage of IGBC, and survival rate. For the quantitative aggregation of the survival results, the correlation between IGBC and survival rate was analyzed according to the hazard ratio (HR), using one of three methods. In studies not reporting the HR or its confidence interval (CI), these variables were calculated from the presented data using the HR point estimate, log-rank statistic or its *p*-value, and the O-E statistic (the difference between the number of observed and expected events) or its variance. 

If those data were unavailable, the HR was estimated using the total number of events, the number of patients at risk in each group, and the log-rank statistic or its *p*-value. Finally, if the only useful data were in the form of graphical representations of survival distributions, the survival rates were extracted at specified times to reconstruct the HR estimate and its variance under the assumption that the patients were censored at a constant rate during the time intervals. The published survival curves were evaluated independently by two authors to reduce variability. The HRs were then combined into an overall HR using Peto’s method [58].

### 2.3. Statistical Analyses

In the present meta-analysis, all data were analyzed and obtained through the Comprehensive Meta-Analysis software package (Biostat, Englewood, NJ, USA). The incidence rates of IGBC after cholecystectomy were investigated from individual studies and analyzed through a meta-analysis. We performed the subgroup analysis based on the type of initial surgery and study, and the study year. The patient’s age and sex were compared between IGBC and overall GBC patients. In this meta-analysis, among fixed and random effect models, for interpretation we used the values of a random-effects model. The heterogeneity between eligible studies was assessed using Q and I^2^ statistics and presented using *p*-values. In addition, the sensitivity analysis was conducted to assess the heterogeneity of eligible studies and the impact of each study on the combined effect. Subgroup analyses were performed based on the type of initial surgery and study, the patients’ age and sex, and the pT stage. The statistical significances between subgroups were evaluated through a meta-regression test. To consider the publication bias, Egger’s test was used. If a significant publication bias was found, the fail-safe N and trim-fill tests were performed to confirm the degree of publication bias. A *p*-value <0.05 was considered significant.

## 3. Results

### 3.1. Selection and Characteristics of Studies

**Figure 1:**
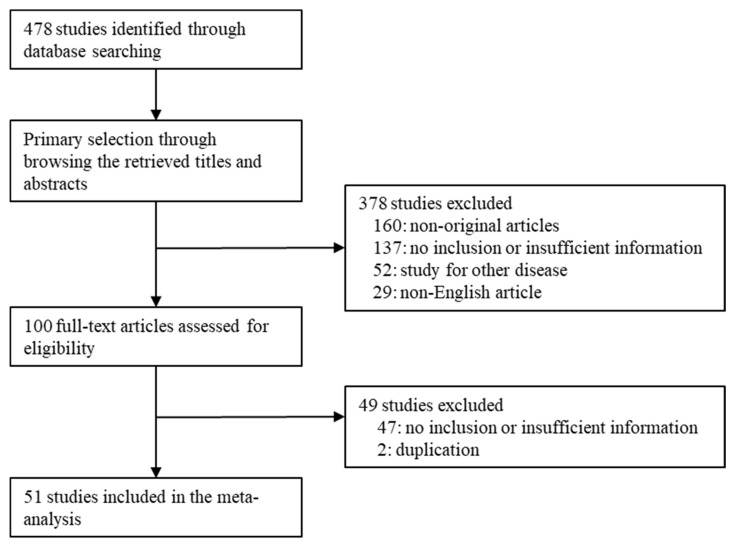
Flow chart of the study search and selection methods.

A total of 478 studies were identified in the database searching for the meta-analysis. Finally, 51 studies were selected according to the inclusion and exclusion criteria. Among the searched studies, 184 studies were excluded due to a lack of sufficient information. In addition, 160 reports were excluded due to being non-original articles. Other remaining reports were excluded for the following reasons: focusing on other diseases (*n* = 52), articles in a language other than English (*n* = 29), and duplicate articles (*n* = 2) (Figure 1). The characteristics of the eligible studies are shown in Table 1.

### 3.2. The Incidence Rate of IGBC after Cholecystectomy

The incidence rate of IGBC after cholecystectomy was 0.6% (95% confidence interval (CI) 0.5–0.8%) in overall patients (Table 2). The incidence rate of IGBC after laparoscopic cholecystectomy was 0.7% (95% CI 0.5–0.9%). The incidence rate of the subgroup using the patients’ registry was significantly lower than that of subgroup using individual hospital data (0.2% vs. 0.7%; *p* = 0.002 in the meta-regression test). However, there were no significant differences of incidence rates between study years.

Next, the patients’ age and sex of IGBC were investigated and compared with overall GBC. Patients with IGBC had a significantly older age than the overall patients (*p* < 0.001 in meta-regression test). The patients’ age of IGBC and overall GBC were 65.291 (95% CI 63.867–66.715) and 52.023 (95% CI 49.208–54.839), respectively (Table 3). The female ratio of IGBC was 69.4% (95% CI 66.0–72.7%). There was no significant difference of female ratio between IBC and overall GBC (*p* = 0.817 in the meta-regression test). The age and female ratio was higher in the subgroup using the registry than in the subgroup using the individual hospital data. The IGBC showed the highest rate in the pT2 stage than in other pT stages (Table 4). The estimated rates of IGBC were 13.0% (95% CI 7.9–20.6%), 34.1% (95% CI 28.3–40.3%), 39.7% (95% CI 34.8–44.8%), 22.7% (95% CI 19.5–26.4%), and 12.5% (95% CI 7.3–20.6%) in the pTis, pT1, pT2, pT3, and pT4 stages, respectively.

### 3.3. Comparison of Prognosis between IGBC and Non-IGBC

**Figure 2:**
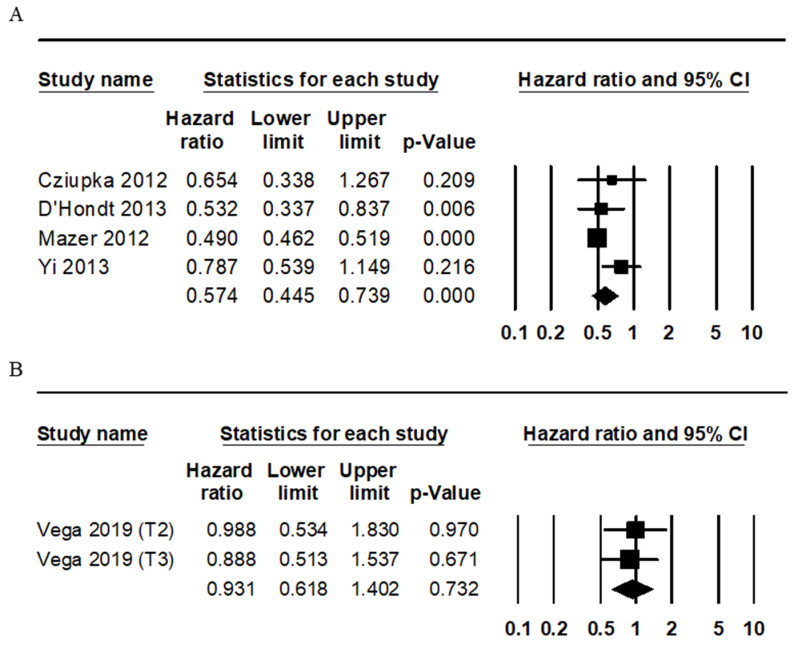
Forest plots for the differences of the survival rates between incidental gallbladder cancer (IGBC) and non-IGBC. (**A**) Overall survival and (**B**) disease-free survival.

The prognosis of IGBC was evaluated by comparing it to non-IGBC. Patients with IGBC had a better overall survival rate than patients with non-IGBC (HR 0.574, 95% CI 0.445–0.739; Figure 2). However, there was no significant difference of the disease-free survival rate between patients with IGBC and non-IGBC (HR 0.931, 95% CI 0.618–1.402).

## 4. Discussion

To the best of our knowledge, the present study is the first meta-analysis to investigate the incidence rate of IGBC and to compare the prognosis of IGBC and non-IGBC patients. Previous studies, including systematic reviews and meta-analyses, have reported IGBC after cholecystectomy. The previous meta-analysis showed the incidence rate and the prevalence of pT stage of IGBC using 26 eligible studies [59]. However, the information for the prognosis of IGBC cannot be obtained in the previous meta-analysis. We also analyzed the incidence rates according to the type of surgery and study and the patients’ age and sex.

Eligible studies of the current meta-analysis showed the information for the combined group with laparoscopic and open cholecystectomy or laparoscopic cholecystectomy alone. Among the eligible studies, data for the incidence of IGBC after open cholecystectomy could not be obtained. In daily practice, laparoscopic cholecystectomy is a gold standard for benign diseases of GB rather than open cholecystectomy. However, the comparison of the incidence rate of IGBC between laparoscopic and open cholecystectomies could not be performed due to no information for open cholecystectomy.

We indirectly compared the incidence rates between patients with laparoscopic cholecystectomy and overall patients. The estimated incidence rates of IGBC were 0.7% and 0.6% in patients with laparoscopic cholecystectomy and overall patients, respectively. The incidence rates ranged from 0.1% to 2.9% in the eligible studies. Eligible studies obtained the information from a single individual hospital or public registry. The estimated incidence rate of the single individual hospital was significantly higher than that of the public registry (0.7% vs. 0.2%). This discrepancy may be caused by the size of the population and different hospitals. In addition, because single individual hospital data may be obtained from tertiary hospitals or training hospital, the ratio of elective cholecystectomy with benign diseases can be lowered.

Unlike the previous meta-analysis, we performed the meta-analysis for the overall and disease-free survival rates of IGBC compared to non-IGBC. However, the information for the disease-free survival rate was only shown in one study [48]. As this study showed the disease-free survival rates divided into pT2 and pT3 stages, the meta-analysis could be performed. However, there was no significant difference of the disease-free survival between patients with IGBC and non-IGBC. In the comparison of overall survival rates, patients with IGBC had a favorable prognosis compared to patients with non-IGBC. However, among eligible studies, some studies demonstrated no significant difference of overall survival rates [12,51]. 

This difference of prognosis between IGBC and non-IGBC may be caused by the difference of the tumor stage at the initial diagnosis (pT stage) in IGBC than in non-IGBC. In the current meta-analysis, IGBCs were detected from pTis to pT4. The highest rate of pT stage was the pT2 stage in IGBC (39.7%, 95% CI 34.8–44.8%). In the current study, patients with IGBC were frequently confirmed to pTis to pT2 stages. The pT3 and pT4 stages were 22.7% and 12.5%, respectively. However, the previous meta-analysis reported that the pT4 stage rate of IGBC was 4.2% (Choi KS 2015). In addition, patients with a lower pT stage had better survival rates than those with a higher pT stage, regardless of preoperative suspicion [12,33]. Thus, the lower pT stage of IGBC may impact the better prognosis of IGBC compared to non-IGBC. Further evaluation for the prognosis of IGBC and non-IGBC will be needed based on the stratification of pT stage. As the extent of pathologic examination can impact the pT stage, further evaluations are needed.

A previous study reported that the preoperative nonsuspicious cases were 50–70% of the overall GBCs [54,60]. For the detection of GBC in the postoperative pathologic examination, the appropriate sections are needed for the suspicious lesion. However, in daily practice, because all portions of GB cannot be evaluated in the microscopic examination, the careful macroscopic examination from expert pathologists may be important. Previous studies suggested the effect of frozen sections on the detection of IGBC [4,36]. 

The intraoperative detection of IGBC with pT2 or higher stages via frozen sections can be useful for the prompt conversion of radical surgery [47]. The rates of pTis and pT1 stages were 13.0% and 34.1%, and the frozen section was not useful for non-mass-forming GBC. Therefore, in pT2 and higher stages, the intraoperative frozen may be useful for infiltrative lesions into the adjacent tissue. Nitta et al. [36] suggested that the examination for the full thickness of the gallbladder through the frozen section could be useful for the detection of IGBC and differentiation between pTis/T1a and others. The most common macroscopic feature is the wall thickening of GBC. As there are limitations on the extent and time of examination of frozen sections, the impact on the detection of IGBC is limited. Frozen sections can be useful for suspicious lesions detected by surgeons [4].

Some limitations in the current meta-analysis exist. First, in this meta-analysis, the pN stage and nodal disease were not analyzed. Laparoscopic cholecystectomy is enough for treatment for IGBC with the pTis to pT1 stage. Therefore, lymph node dissection could not be performed in many cases with IGBC. Second, by definition, missing cases in the preoperative radiologic examination should be considered for non-IGBC. However, the detailed information for preoperative radiologic findings could not be found in the eligible studies. Based on this limitation, the incidence rate of IGBC may be lower. Third, the present study was not performed for the subgroup analysis for tumor types, such as lymphoma and squamous cell carcinoma, due to the very low incidence of other tumor types. Fourth, the detailed comparisons between IGBC and benign diseases could not be performed due to insufficient information on eligible studies. However, IGBC frequently occurred with older age and male subjects, compared to benign diseases. Fifth, the impact of histologic examination on the detection rate of IGBC could not be investigated due to insufficient information of the examined section or extent. Sixth, the impact of pT stages of IGBC on survival rates could not be obtained due to insufficient information. Seventh, subgroup analysis based on the types of benign diseases, such as acute/chronic cholecystitis, could not be obtained due to insufficient information.

## 5. Conclusions

In conclusion, IGBC was found in 0.6% of cholecystectomy with benign diseases. IGBCs had higher rates in the pT1 and pT2 stages and frequently occurred in older patients. In addition, patients with IGBCs had a better prognosis than those with non-IGBCs. To detect IGBC after cholecystectomy, a sufficient examination for histology is needed in the pathologic examination.

## Figures and Tables

**Table 1:** Main characteristics of the eligible studies.

First Author, Year	Location	Study Year	Using Registry	Initial Surgery	No of Patients		Tumor Stage					
					Total	IGBC	Tx	Tis	T1	T2	T3	T4
Ahn 2016	Korea	1998−2014		ND	4629	73						
Aoki 2002	Japan	1990−1999		LC	990	11		5		6		
Apodaca-Rueda 2017	Brazil	2010−2015		ND	893	13						
Basak 2016	Turkey	2009−2013		LC/Open	1747	4				3	1	
Braghetto 1999	Chile	1992−1998		LC	6500	15			4	5	6	
Cavallaro 2012	Italy	1998−2008		LC	1480	9		1	4	4		
Chan 2003	Taiwan	1992−2000		LC	1825	11			3	4	3	1
Charfi 2018	Tunisia	2003−2016		LC/Open	20,584	155			18	68	36	33
Choi 2009	Korea	2002−2007		LC	3145	33			12	17	4	
Cziupka 2012	Germany	2001−2009		ND	ND	12			3	5	3	1
D’Hondt 2013	Belgium	1998−2008		LC/Open	ND	45						
Dorobisz 2016	Poland	1990−2014		LC/Open	7314	64						
Duzkoylu 2015	Turkey	2005−2013		LC	8698	15		3	2	5	3	
Ferrarese 2013	Italy	2008−2012		LC	508	7				5	2	
Firat 2019	Turkey	2015−2017		LC/Open	1112	7		1	3	3		
Genc 2011	Turkey	1999−2010		LC	5164	5		1	1	1	2	
Geramizadeh 2018	Iran	2010−2016		LC/Open	4872	18			10	8		
Glauser 2011	Switzerland	ND	Yes	LC/Open	30,960	69		2	14	34	14	5
Goussous 2018	USA	2000−2013		LC/Open	5796	26						
Gulwani 2015	India	2001−2013		LC	2926	23			5	14	4	
Horkoff 2019	Canada	2001−2015	Yes	LC/Open	11,4951	129						
Ioannidis 2013	Greece	1992−2001		LC/Open	1536	14			5	6	3	
Jha2018	India	2014−2016		LC/Open	4800	20			18	2		
Kalita 2013	India	2009−2012		LC/Open	4107	18		1	7	10		
Kim 2010	Korea	1997−2008		LC/Open	2607	26		1	6	17	2	
Koppatz 2018	Finland	2010−2012		LC/Open	2034	10						
Kwon 1997	Korea	1990−1996		ND	527	10			5	3	2	
Kwon 2008	Japan	1992−2004		LC	1793	38			20	17	1	
Lundgren 2018	Sweden	2007−2014	Yes	LC/Open	36,010	213	23	14	41	72	51	12
Martins-Filho 2015	Brazil	2007−2010		LC/Open	2018	10						
Mazer 2012	USA	1984−2008		ND	ND	443						
Mitrovic 2010	Bosnia et al.	ND		LC/Open	3007	21						
Mori 1997	Japan	1991−1995		LC	456	13			9	3	1	
Nitta 2018	Japan	2009−2017		LC/Open	529	8		2	1	3	2	
Panebianco 2013	Italy	2003−2011		LC	1188	6			1	2	3	
Patel 2016	UK	2008−2013		LC/Open	4027	7		1	2	2	2	
Pitt 2014	USA	2005−2009	Yes	LC/Open	91,260	170						
Sarli 2000	Italy	1992−1999		LC/Open	2300	20		1	6	4	9	
Shimizu 2006	Japan	1991−2004		LC	1195	10			4	5	1	
Solaini 2014	UK	2005−2012		LC/Open	864	7						
Talreja 2016	Pakistan	2005−2015		LC	964	11		1	4	6		
Tantia 2009	India	2004−2007		LC	3205	19		8	8	3		
Tatli 2017	Italy	2013−2016		LC/Open	341	7			3	4		
Tian 2015	China	2002−2012		LC	7582	69			22	16	13	18
Utsumi 2017	Japan	2008−2015		LC	352	8			3	4	1	
Vega 2019	USA	1999−2016		ND	ND	11						
Xu 2013	China	1993−2011		LC	8005	36			16	11	9	
Yamamoto 2005	Japan	1991−2003		LC	1663	9		1	3	5		
Yi 2013	China	1992−2009		LC/Open	14073	38			14	4	13	7
Zhang 2015	China	1999−2007		LC	11574	28		4	9	8	5	2
Zhu 2015	China	2000−2010		LC	4014	29			11	14	4	

No, number; IGBC, incidental gallbladder cancer; Tis, in situ; ND, no description; LC, laparoscopic cholecystectomy.

**Table 2:** The estimated rates of incidental gallbladder cancer after cholecystectomy.

Subgroup	Number of Subsets	Fixed Effect [95% CI]	Heterogeneity Test (*p*-Value)	Random Effect [95% CI]	Egger’s Test (*p*-Value)
Overall	47	0.005 [0.005, 0.005]	<0.001	0.006 [0.005, 0.008]	0.070
Laparoscopic	21	0.007 [0.007, 0.008]	<0.001	0.007 [0.005, 0.009]	0.517
Using registry	4	0.003 [0.002, 0.003]	<0.001	0.002 [0.001, 0.005]	0.499
Individual	43	0.007 [0.007, 0.008]	<0.001	0.007 [0.006, 0.008]	0.294
Included 1990’s	19	0.007 [0.006, 0.007]	<0.001	0.007 [0.004, 0.010]	0.972
Included 2000’s	21	0.004 [0.004, 0.004]	<0.001	0.006 [0.004, 0.008]	0.253
after 2010 year	6	0.006 [0.005, 0.008]	<0.001	0.007 [0.004, 0.012]	0.198

CI, Confidence interval.

**Table 3:** Comparisons of age and female ratio between incidental and non-incidental gallbladder cancers.

Subgroup	Number of Subsets	Fixed Effect [95% CI]	Heterogeneity Test (*p*-Value)	Random Effect [95% CI]	Egger’s Test (*p*-Value)
Age					
IGBC	41	70.239 [69.945, 70.534]	<0.001	65.291 [63.867, 66.715]	<0.001
Laparoscopic	19	69.234 [68.703, 69.765]	<0.001	64.179 [61.280, 67.077]	<0.001
Using registry	3	71.613 [71.208, 72.017]	0.083	71.149 [69.890, 72.408]	0.524
Individual	38	68.695 [68.265, 69.124]	<0.001	64.629 [62.801, 66.458]	<0.001
Overall	17	49.797 [49.770, 49.824]	<0.001	52.023 [49.208, 54.839]	0.199
Laparoscopic	4	48.289 [48.048, 48.529]	<0.001	49.014 [43.925, 54.103]	0.938
Individual	17	49.797 [49.770, 49.824]	<0.001	52.023 [49.208, 54.839]	0.199
Female ratio					
IGBC	41	0.695 [0.668, 0.721]	0.113	0.694 [0.660, 0.727]	0.980
Laparoscopic	19	0.658 [0.607, 0.705]	0.671	0.658 [0.607, 0.705]	0.333
Using registry	3	0.735 [0.691, 0.775]	0.001	0.761 [0.628, 0.857]	0.588
Individual	38	0.673 [0.638, 0.706]	0.744	0.673 [0.638, 0.706]	0.147
Overall	23	0.683 [0.681, 0.685]	<0.001	0.689 [0.659, 0.717]	0.891
Laparoscopic	6	0.661 [0.652, 0.669]	<0.001	0.635 [0.584, 0.683]	0.187
Individual	23	0.683 [0.681, 0.685]	<0.001	0.689 [0.659, 0.717]	0.891

CI, Confidence interval; IGBC, incidental gallbladder cancer.

**Table 4:** The estimated rates of tumor stages of incidental gallbladder cancer after cholecystectomy.

Subgroup	Number of Subsets	Fixed Effect [95% CI]	Heterogeneity Test (*p*-Value)	Random Effect [95% CI]	Egger’s Test (*p*-Value)
Tumor stage					
pTis	17	0.125 [0.094, 0.163]	0.002	0.130 [0.079, 0.206]	0.853
pT1	34	0.300 [0.270, 0.331]	<0.001	0.341 [0.283, 0.403]	0.022
pT2	37	0.395 [0.364, 0.426]	0.001	0.397 [0.348, 0.448]	0.957
pT3	27	0.230 [0.203, 0.260]	0.253	0.227 [0.195, 0.264]	0.311
pT4	8	0.156 [0.126, 0.191]	<0.001	0.125 [0.073, 0.206]	0.255

CI, Confidence interval.
